# Proceedings of the Georgia State Dental Society

**Published:** 1881-09

**Authors:** 


### ARTICLE II.

### *Proceedings of the Georgia State Dental Society.*

SAVANNAH, GA. May 10, 1881.

The Thirteenth Annual Session of the Georgia State Dental Society convened in the Dining Room of Screven House, at eleven o'clock, A. M., Dr. R. W. Thornton, President, in the Chair.

Rev. Mr. Kerr offered a prayer, after which he addressed the Society on the subject of Charity.

Dr. Hopps then delivered an address of welcome to the members of the Society, throwing open the gates of the city and promising all a good time, which was fully verified by subsequent events.

Dr. W. W. Ford, of Macon, replied to Dr. Hopps on behalf of the Society, and thanked him for the kind expressions of welcome.

A few minutes recess allowed that those present might have an opportunity to pay their dues.

The Executive Committee recommended for active membership J. P. Huntley, LaGrange; B. H. Patterson, Baxley;

Allen Brown, Blackshear; Thos. G. Cowardin, Savannah; M. G. Little, Crawford.

On motion, the Secretary was authorized to cast the vote of the Society, which resulted in the election of each in regular order.

Dr. Marks, of Milwaukie, was invited to a seat on the floor.

Roll called and the following noted present: E. Parsons, Savannah; H. A. Lowrance, Athens; A. C. Ford, Fernandina, Fla; L. D. Carpenter, Atlanta; G. W. McElhaney, West Point; J. L. Fogg, Barnesville; W. W. Ford, S. B. Barfield, Macon; A. G. Bouton, D. Hopps, Savannah; R. W. Thornton, Calhoun, Ga; J. H, Coyle, Thomasville; J. P. Holmes, W. R. Holmes, Macon; W. C. Wardlaw, Augusta; R. A. Holliday, Atlanta; G. W. H. Whitaker, Sandersville; J. P. Huntley, LaGrange; Allen Brown, Blackshear; Thomas G. Cowardin, Savannah; M. G. Little, Crawford; B. H. Patterson, Baxley.

The time having arrived, the President delivered the Annual Address.

The minutes of previous meeting were read and adopted.

The President appointed a Committee on Clinics consisting of Drs. Hopps, A. G. Bouton, and G. W. H. Whitaker.

Adjourned to three o'clock, P. M.

---

### AFTERNOON SESSION.

*Three o'clock, p. m.*

The Society was called to order, President Dr. R. W. Thornton in the Chair.

The first order of business was hearing the reports of Standing Committees.

*Physiology and Etiology*—W. C. Wardlaw, W. R. Holmes.

Dr. Wardlaw read a paper on the last named, reciting a case in practice, which was received and opened for discussion.

Dr. Hopps asked if the gums were in an inflamed condition. Answer—No, they were not.

Dr. Parsons—I have been called upon several times where teeth had been extracted by physicians, and in several cases the patient was so weak that he could not raise his hands. First case was bleeding from extraction of lower molar. I put my finger on the part and held it for two hours; don't know whether the pressure or the magnetic action in the fingers stopped the bleeding. Second case—held fingers in the parts for three hours, which had the desired result. About three weeks ago a lady called at my office and wanted some teeth extracted, saying that she came very near bleeding to death from extraction. I used my local anæsthetic and took out eight teeth, and she said that she only felt pain in the last. I think there is an abundance of saline matter in the hemorrhage blood which can be detected with the microscope, or better with the spectroscope. I sometimes dip my finger in the blood, when suspected, and taste it, and the difference is very marked.

Dr. Ricks—I extracted left inferior cuspid which presented no appearance of profuse hemorrhage at the time, but the patient returned, stating that considerable blood had been lost. I used per. chloride of iron by saturating cotton with it, and packing the cavity. The patient returned again, tooth still bleeding. I again used the iron but the bleeding did not cease, and he consulted an old man who professed to heal by a secret art, and by the next day it had ceased, and the old man received credit for the cure.

Dr. W. W. Ford had used the per. chloride of iron by packing the cavity with cotton saturated with it and putting on compress.

Treated a patient, (a young man,) by packing the cavity with cotton saturated with iron, applying compress, which caused the hemorrhage to cease, but when he returned home they put him to plowing and the bleeding commenced again; this was repeated several times. Have used cotton saturated with nutgalls covered with sandarac varnish, successfully. Would use, where all the teeth had been extracted and bleeding from the sockets, fill the sockets with cotton saturated with tannic acid and take a plaster impression, removing the cup and leaving the plaster impression in the mouth until the bleeding ceased.

Dr. Parsons, in reply to an enquiry, stated that the predisposing cause of hemorrhage was an excess of blood and a want of coagulum. The inferior teeth are the ones predisposed to hemorrhage.

Dr. W. L. Smith related a case where he had used tinct. muriate of iron without success; afterwards used nitrate of silver, which caused the bleeding to cease; thought burnt alum good.

Dr. Wardlaw used linen strips sprinkled with tannin and saturated with sandarac varnish. Where there is more than one socket bleeding, commences at posterior and continues to fill them with the strip, afterwards making a compress. I think that nervous or excitable persons are predisposed, or mental impressions have a great deal to do with the hemorrhage.

Dr. Parsons—It generally occurs in persons of florid complexion.

Dr. W. W. Ford—I have never seen a case of hemorrhage in a decided brunette; believe that mental impressions influence the flow of the blood.

Dr. Hopps—As there are different size streams in different sections of the country, so it is with the teeth; some are supplied by larger arteries than others, and consequently larger arteries are ruptured. I never saw excessive bleeding from more than one socket in the same mouth.

Subject passed.

Dental Pathology and Surgery was called and passed.

Dental Histology and Microscopy was called and passed subject to recall.

Chemistry and Therapeutics called.

The Chairman of Committee on Clinics stated that Dr. J. P. Huntley had consented to give a Cl[illegible]ght o'clock to-morrow morning.

Dr. Whitaker moved that the Society hold a night session for the purpose of correcting the list of Dentists practicing in the State. Motion prevailed.

Dr. Ford then read a paper on Chemistry and Therapeutics in Dentistry, which was received, and discussion postponed. Adjourned till eight o'clock, P. M.

---

NIGHT SESSION.

*Half-past Eight o'clock, p. m.*

The Society was called to order. President R. W. Thornton in the Chair. G. W. H. Whitaker, Secretary *pro tem.*

After correcting the list of Dentists, the Society adjourned to meet at nine o'clock in the morning.

---

SECOND DAY—MORNING SESION.

*Half-past Nine o'clock, a. m.*

The Society was called to order. President R. W. Thornton in the Chair.

The Executive Committee recommended Drs. S. M. Roach and J. G. Keller for active membership, when they were balloted for and elected as such.

Dr. Wardlaw objected to the Executive Committee making any further report, as the discussion of Dr. Ford's paper was the next business.

The Chair ruled that the Executive Committee could report at any time, which was sustained.

The Executive Committee reported their action in regard to further legislation, suggesting that each member see their immediate representative and get his co-operation for the passage of an Exemption Act.

Dr. Hopps read as part of the attached report a memorial, with bill, which he had prepared for the Legislature. This elicited some discussion concerning the best mode of securing the co-operation of the members of the Legislature. The report was then received.

Dr. Holmes moved that the Executive Committee be authorized to have a sufficient number of Dr. Hopps' memorial printed and mailed to each Representative. Carried.

Judge John W. Hopkins, of Thomasville, was tendered a seat on the floor.

Dr. N. A. Williams was unanimously elected an active member of the Society.

Dr. J. W. Daniels, of Darlat, was recommended for membership by the Executive Committee, and unanimously elected an active member.

Operative Dentistry called.

Dr. Hopps read a paper entitled Operative Dentistry, which was received and adopted.

Dr. A. C. Ford said he had used Fletcher's Cement in the way Dr. Hopps used the gutta percha, in cases where the gold or amalgam would be likely to show.

Dr. Hopps stated that he did not use this method where the walls of the cavity were broken away.

Dr. Holmes uses pellets or rope of Japanese bibulous paper, saturated with chloride of zinc, pressed up under the gum in labial cavities where the rubber dam could not be used, without trouble; uses it in the same way for inferior incisors.

Dr. Hopps was never successful with such cavities until the rubber dam came into use.

Dr. Smith said that he used the ligature in the same way Dr. Hopps mentioned, but carried it around twice, or two wraps.

Dr. Adair—Dr. Hopps spoke of his use of soft and cohesive gold alternately. I would like for Dr. Hopps to explain how he can use cohesive gold, which has a tendency to roll away from the walls, and soft, which presses out.

Dr. Hopps—I use a soft pellet first and condense it; next, cohesive gold and weld it.

Dr. Bouton—Dr. Hopps does not make a distinction between cohesive and soft gold. Perfectly soft foil cannot be made cohesive by annealing. Pure gold is adhesive.

Dr. W. W. Ford—I take issue with Dr. Bouton. I don't think all pure gold is cohesive.

Dr. Adair—I have my doubts about using cohesive and soft foil in the same cavity and making a condensed filling. I use Slayton's Felt Foil.

Dr. W. W. Ford—I use Caulk's Diamond Point Pellets before filling with amalgam.

Dr. Hopps—I once put two teeth—one filled with soft foil, and the other with cohesive—into a solution of nitrate of silver. When taken out, the cohesive filling was discolored, while the soft filling was not the least discolored.

Dr. Wardlaw—I have been listening for some satisfactory reason for Dr. Hopps' combination filling. I don't think that gutta percha is better than some of the other plastics. I use Metcalf's Cement, which strengthens the walls, and is a non-irritant; use the smallest amount of metal possible to prevent thermal changes; think it a good deal of trouble to get the gutta percha wafer in the cavity as described.

Dr. Wardlaw asked Dr. Hopps to give his definition of Dr. Arthur's Double V.

Dr. Bouton—Before Dr. Arthur's death he gave up his original V shape space and made the space larger at the neck, and not near the cutting edge. To cut a tooth through from the cutting edge up to the gum, and make a V shape up to the gum, I am very doubtful about, as it will cause trouble. Teeth separated in this way, and filled, will give way.

Dr. S. B. Barfield related a case in practice: A lady patient, previous to coming to him, had all her teeth filled with gold by a good operator, but the fillings had failed. She had consulted Dr. Parsons, and he refilled with gutta percha the majority of the cavities. She had suffered a great deal with her teeth up to the time of the change of the filling material. Since then she has been doing very well, and the fillings are preserving the teeth.

Subject passed.

Mechanical Dentistry called and passed.

The Executive Committee recommended Dr. H. J. Royall for reinstatement, and Dr. Albert Lefler for active membership in the Society, and they were unanimously elected as such.

Dental Education and Literature called.

Dr. A. C. Ford stated that he had not prepared any paper on the subject; spoke in favor of a National Dental College, to be endowed; thought that students were graduated too easily, yet it was not worse with Dentists than the other professions; the diploma of D. D. S. is not recognized in Europe.

Dr. Parsons—I have doubts about such a College as Dr. Ford speaks of being recognized in Europe. It is not so with Medical Colleges; they are recognized,

Dr. Wardlaw—We have an enterprise in Dental Literature; that is *The Dental Luminary.* The proprietors should be encouraged, as it is calculated to do more good towards the education of the Dentists than any other medium.

Dr. Hopps—I think the profession is progressing, and that the Colleges are doing the best they can under the circumstances.

Dr. Holmes thought Dental Graduates would compare favorably with the graduates in other professions.

Subject passed.

A paper on "The New Departure," by Dr. J. H. Coyle, was received and opened for discussion.

Dr. Fogg—Gold is the best material to fill teeth with, but there are teeth which are not compatible with metal; these are better filled with gutta percha.

Dr. A. G. Bouton read an interesting and instructive essay on the subject of treating the Dental pulp when exposed in its various stages, and the successful preservation of the same, giving practical daily experience, etc.

An article on the subject of "Capping Nerves," by Dr. J. M. Mason, was read, detailing his *modus operandi* and success in performing this operation, he having great success by his treatment.

A motion to receive these papers, and that they be opened for discussion, was carried.

A motion to adjourn was made and carried.

---

THIRD DAY—MORNING SESSION.

*Half-past Eight o'clock, a. m.*

Dr. Thornton, President, in the Chair.

The Executive Committee recommended Dr. W. L. Smith, of Cochran, and Dr. T. J. Key, of McVille, for active membership, and they were balloted for and elected.

Dr. McElhaney read a paper on the "Denuding of the Teeth."

On motion, the paper was received, and discussion postponed.

The Executive Committee submitted the following resolution, which was adopted.

*Resolved*, That a committee of one from each judicial district in the State be appointed, whose duty it shall be to ascertain the name and address of every Dentist in his district, and also whether said Dentists are practicing in accordance with the requirements of the laws of the State of Georgia, and to report to the Chairman of the State Executive Committee.

The resolution was adopted, and the Executive Committee authorized to make the appointment.

Adjourned to half-past eight P. M.

---

EVENING SESSION.

*Half-past Eight o'clock, p. m.*

Dr. Samuel A. White, of Savannah, was recommended by the Executive Committee for active membership.

On motion, the Secretary cast the ballot which resulted in his election.

Drs. W. W. Ford, W. C. Wardlaw and D. L. Ricks were appointed to draft resolutions of thanks.

The following resolution was offered by Dr. W. W. Ford of Macon:

*Resolved*, That when this session of the Society adjourns, it adjourns to meet on the second Tuesday in May, 1882, in the city of Macon, Ga.

The resolution was adopted.

Dr. Adair related a case of a young man who had received a blow on the right superior central incisor, breaking it. It did not give any trouble for three or four years. It recently abscessed; is loose; had treated abscess, and now wants to know how the tooth can be saved and made firm.

Dr. A. C. Ford suggested using platina wire in the root, and filling the tooth with some of the phosphates.

Dr. S. A. White thought it advisable to get the tooth out as soon as possible, as there could be no union of the dentine, that is, if there is a fracture; the periosteum is no doubt destroyed, and the portion of the root is a foreign body.

Dr. A. C. Ford—I cannot give an opinion without seeing the patient, as it has been preserved for several years, and the patient still wishes to retain it.

Dr. McElhaney's paper was re-read.

Dr. Ford thinks it is not a mechanical injury, but caused from some eruptive disease during the formation of the permanent teeth, or perhaps typhoid fever.

Dr. J. P. Holmes thinks that the denuding is caused by denuding or drawing away of the gums, thereby exposing the roots of the teeth.

Dr. Thornton—The case that Dr. McElhaney describes is what Harris calls atrophy. I do not think it is a disease, but the result of disease.

Dr. A. C. Ford—Dr. McElhaney describes atrophy. I usually cut out the defect with a corundum disk, finishing with a disk of rubber and corundum; where the defect is deep I cut it out and fill, making as little display as possible.

Dr. White—I think there are two characteristics—one showing decay, and where it shows decay should fill it, but where there is a smooth groove, don't think it will do any good to fill, as it will leave a raised filling.

Dr. Parsons—As I understand the word, denuding means uncovering. There are two kinds of abrasion—chemical and mechanical. I believe it is a want of silicate of lime; it is very slow in its progress.

Dr. Wardlaw—Evidently the case Dr. McElhaney mentions is congenital. Dr. Ford's case is described by Harris as abrasion. It can only take place by two means, that is, by chemical and mechanical; don't think any good can be done by filling; think that alkalies are used successfully in such cases.

Dr. Ford—I believe it is caused by acidity of the saliva.

Dr. Allen Brown exhibited the temporary teeth of a child where the central and lateral incisors were united; also a molar tooth which was taken from the place usually occupied by the central incisors, in about four months after the centrals were erupted.

Adjourned to eight o'clock, A. M., 13th.

---

### FOURTH DAY—MORNING SESSION.

*Eight o'clock, a. m.*

Dr. Thornton, President, in the Chair.

The Executive Committee reported favorably on the application of T. T. Bessellieu, of Savannah, for active membership, and on motion, the Secretary cast the ballot of the Society, which resulted in his election.

The Executive Committee recommended the beneficiaries be conferred on the following: Mr. W. J. Flanders, University of Tennessee; Mr. G. J. Ford, to Baltimore College of Dental Surgery, and W. S. Jordan to Philadelphia Dental College.

On motion, the report was adopted and the Secretary was instructed to furnish certificates to those elected.

The Committee appointed to draft resolutions of thanks, submitted the following:

*Resolved*, That the most sincere and unfeigned thanks of this Society are eminently due, and are hereby cordially tendered to our professional brethren of this city, who are members of this Society, for their unprecedented kindness and liberality to us as a Society and as individuals, during the whole of this delightful session in their beautiful city of Savannah.

*Resolved*, That we tender our thanks to the proprietors of the Screven House, and also to the various railroads that have given us reduced rates, for their kindness and liberality in doing so.

All of which is respectfully submitted.

The report was adopted.

Dr. Carpenter related his experiments with the different amalgams in use; thinks that the difficulty with most amalgams is in the melting—the tin is burned up by the excessive heat of the silver.

Dr. Coyle—I don't think any of the tests out of the mouth can be relied upon, as they lose sight of the heat.

Dr. Carpenter's report was received with thanks.

Dr. Lowrance made a motion that the Society elect officers at half-past ten, which was amended by inserting eleven o'clock, which motion prevailed.

A motion to reconsider was carried, and the Society then went into an election of officers, with the following result:

W. W. Ford, President; A. L. Smith, 1st Vice-President; D. Hopps, 2d Vice-President; L. D. Carpenter, Corresponding Secretary; R. A. Holliday, Recording Secretary; H. A. Lowrance, Treasurer.

*Executive Committee*—J. H. Coyle, A. G. Bouton, L. D. Carpenter, S. B. Barfield and G. W. McElhaney.

The officers were requested to take the seats to which they had been elected, without the usual formalities, which request was complied with.

Dr. Ford, the newly elected President, on assuming the duties of the office to which he had been elected, made a few appropriate remarks, asking the assistance of each member to unite with him in discharging his duties successfully, and thanking all for the honor conferred on him.

Dr. Thornton, on vacating the Chair which he had so capably filled, also made a few appropriate remarks, re turned thanks for courtesies, etc.

A motion was made for the Treasurer to read his Report, which was carried, and the Report was received and the Secretary instructed to spread it upon the minutes.

A motion was made and carried that Dr. Bouton be re quested to read again his essay on Capping of Pulps, which he did, and a lengthy discussion ensued.

Dr. Parsons made a motion that Dr. Whitaker be authorized to have enough extra copies of the list of Dentists in this State to furnish each member with one.

Dr. McElhaney moved to amend by adding that each member of this Society in good standing, be so marked in the list.

A substitute was offered that Dr. Holmes be authorized to have a list of the Dentists in this State printed, and that each member of this Society in good standing, be so marked.

Dr. Wm. Noble was elected as an active member, dues to be paid to the Treasurer on his return home.

The President appointed the following delegates to the American Dental Convention:

J. P. Huntley, R. A. Holliday, J. L. Fogg, D. Hopps, G. W. H. Whitaker and J. P. Holmes.

Southern Dental Association:

J. P. Huntley, G. W. H. Whitaker, W. C. Wardlaw, D. Hopps, R. A. Holliday, R. B. Adair and J. L. Fogg.

National Association:

B. H. Patterson, J. P. Holmes and R W. Thornton.

On motion, the Secretary was instructed to notify Dr. T. J. Key of his election as an active member of the Society.

Dr. Whitaker made a motion that Dr. J. P. Holmes assist the Secretary in arranging the minutes and papers of the Society during this session, for publication in pamphlet form, and that one hundred or more copies be printed.

Motion of Dr. Lowrance to authorize the Secretary to draw on the Treasurer for the amount necessary to defray the expense of printing the minutes. Carried.

The Society having transacted all the business of this session, adjourned to meet on the second Tuesday in May, 1882, at Macon, Georgia.

R. A. Holliday, *Secretary.*

